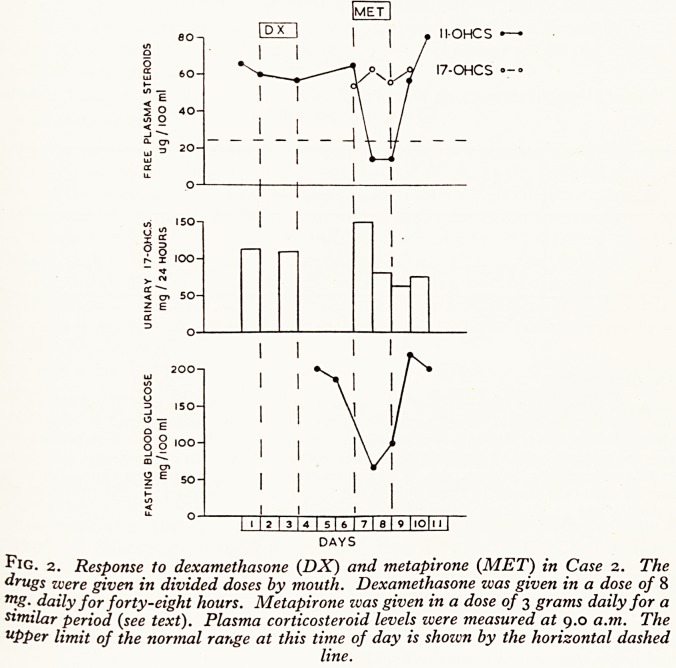# Adrenocortical Hyperfunction and Oat-Cell Carcinoma of the Bronchus

**Published:** 1964-01

**Authors:** D. Mattingly, P. M. Keane, C. F. McCarthy, A. E. Read

**Affiliations:** From the Departments of Medicine and Clinical Pathology, the University of Bristol and the Postgraduate Medical School of London; From the Departments of Medicine and Clinical Pathology, the University of Bristol and the Postgraduate Medical School of London; From the Departments of Medicine and Clinical Pathology, the University of Bristol and the Postgraduate Medical School of London; From the Departments of Medicine and Clinical Pathology, the University of Bristol and the Postgraduate Medical School of London


### ADRENOCORTICAL HYPERFUNCTION AND OAT-CELL CARCINOMA OF THE BRONCHUS

BY

D. MATTINGLY, P. M. KEANE, C. F. MCCARTHY, and A. E. READ

*From the Departments of Medicine and Clinical Pathology, the University of Bristo and the Postgraduate Medical School of London*

The association of increased adrenocortical activity with carcinoma in certain site notably the bronchus, has been increasingly recognised in recent years (Allott Skelton, 1960; Bagshawe, 1960). About fifty cases have been reported to date. It possible, however, that the association is not as rare as this figure would suggest, sinc a number of these patients have not had the clinical features associated with Cushing syndrome.

Two further cases of oat-cell carcinoma of the bronchus associated with adren cortical hyperfunction are reported here. The results of our adrenal function studi in these patients support the view that this association is not fortuitous, but is due the secretion of a corticotrophin-like substance by the tumour cells.

*Methods*

The main free plasma 11-hydroxycorticoid in human plasma is cortisol (hydr cortisone), although corticosterone is also present in much smaller amount (Bush Sandberg, 1953). Free plasma 11-hydrocorticoid (11-O.H.C.S.) levels were estimate by the fluorimetric method previously described by Mattingly (1962). A separat estimate of the free plasma corticosterone was also made. The flourimetric metho allows one to measure endogenous cortisol production when the patient is being treate with prednisone, which does not fluoresce. Free plasma 17-hydroxycorticostero (17-O.H.C.S.), urinary 17-oxo and 17-oxogenic steroids, and urinary tetrahydro-11 deoxycortisol (tetrahydro S) were also extimated.

### CASE REPORT

*Case 1.*

M. G., a woman of 63, was admitted to hospital in September 1961 with a history of men disturbance and a cough for three months. She had smoked at least 20 cigarettes a day 40 years.

On admission she was agitated, verbose, and disorientated. There was generalised muscu wasting and weakness, more marked in the proximal muscle groups. All her reflexes we either diminished or absent, and the plantar reflexes were flexor. There were no senso abnormalities and the fundi were normal. A hard lymph node was palpable in the rig supraclavicular fossa and there were signs of consolidation in the right upper lung. The li was enlarged 3 cms. below the right costal margin. Her blood pressure was 160/90 and mark glycosuria, but no ketonuria, was found on routine ward testing.

*Investigations*

Chest X-ray showed collapse and consolidation of the right upper lobe. Biopsy of the rig supraclavicular lymph node revealed tumour tissue with the microscopic appearance of oa cell carcinoma. No definite bony metastases were seen in skeletal X-rays, but minimal rar faction of the pelvis was reported. The pituitary fossa was normal in size.

A leuco-erythroblastic anemia was present and the bone marrow was infiltrated with mali nant cells. Repeated E. E. G.'s showed a moderately severe generalised abnormality, the reco being dominated by moderate amplitude theta activity at 5 c.p.s. which was slightly reduc by visual attention. An E.M.G. of the left deltoid revealed no abnormality. The C.S.F. was normal and an air encephalogram showed only some cerebral atrophy.

The fasting blood glucose level was 155 mg. per 100 ml. with a diabetic glucose tolerance curve. The blood urea was 40 mg. per 100 ml., and hypokalaemic alkalosis was present (potassium 2·3, bicarbonate 34, sodium 138, and chlorides 96 mEq per litre). Whilst taking a normal ward diet without supplements, the urinary excretion of potassium on two consecutive days was 43 and 44 mEq. per day respectively. When her potassium intake was increased to 200 mEq. a day the plasma potassium concentration rose within forty-eight hours to 3·8 mEq. per litre. This intake was maintained until shortly before her death and her plasma potassium concentration remained within the normal range, fluctuating between 3·6 and 4·8 mEq. per litre. An E.C.G. on admission was not suggestive of hypokalaemia but showed non-specific changes compatible with myocardial ischaemia.

*Adrenal function tests*

Free plasma corticosteroid levels were very high and ranged from 55 to 74 μg. per 100 ml. The normal diurnal rhythm was absent. The cortisol secretion rate was 418 mg. per twenty-four hours (Dr. C. L. Cope). The urinary 17-oxogenic steroids were also markedly elevated, ranging from 97 to 134 mg. per twenty-four hours (Table I).

TABLE I

Adrenal Function Tests and Plasma Electrolytes.

| | Case 1 | Case 2 | Normal Range |
|---|---|---|---|
| *Urine* (mg. per 24 hours) | | | |
| cortisol secretion rate | 418 | — | 5–25 |
| 17-oxogenic steroids | 97–134 | — | 5–20 |
| 17-hydroxycorticoids | — | 113 | 5–20 |
| 17-oxo steroids | 21–23 | — | 5–20 |
| tetrahydro-11-deoxycortisol | 3·7 | — | <1 |
| *Plasma* (μg. per 100 ml.) | | | |
| free 11-O.H.C.S. 9. am. | 64·5–73·2 | 60·1–66·1 | 6–24 |
| 6 p.m. | 54·7 | 66·6 | 3–17 |
| midnight | 55·4 | — | 0–6 |
| free 17-O.H.C.S. 9 a.m. | 55·6–73·9 | 53·5 | 6–24 |
| *Plasma Electrolytes* (mEq. per litre) | | | |
| potassium | 2·3 | 2·0 | 3·6–4·8 |
| bicarbonate | 34 | 35 | 25–30 |
| sodium | 138 | 142 | 136–148 |
| choloride | 96 | 80 | 96–106 |

*Responses to metapirone*

Metapirone was given by mouth for seven days. The initial dose was 500 mg. four-hourly, but this was increased to one gram four-hourly for the last thirty-six hours. Changes in plasma corticosteroid levels, urinary 17-oxogenic steroids and fasting blood glucose levels are shown in Figure 1.

The free plasma 11-O.H.C.S. level fell from 67 to 20 μg. per 100 ml., and significant falls were also seen in the free plasma 17-O.H.C.S. level and in the urinary 17-oxogenic steroids. Urinary tetrahydro S levels, on the other hand, rose from 3·7 to 82 mg. per twenty-four hours. The fasting blood glucose level fell from 265 to 116 mg. per 100 ml. when the dose of metapirone was increased to 750 mg. every four hours.

The blood pressure fell from 170/110 to 130/70 during metapirone administration, but rose to 160/100 four days after stopping the drug. There was a marked but transient improvement in her mental state two days after stopping metapirone. She was able to recognise members of her family and have lucid conversations with them which she had not been able to do for some weeks.

Her condition deteriorated fairly rapidly after this and her haemoglobin level fell to 6·7 g per 100 ml. She developed widespread purpura and died in coma one month after admissio to hospital. The free plasma 11-O.H.C.S. level rose to 115 $\mu$g. per 100 ml. a few hour before death.

FIG. 1. *Response to metapirone in Case 1. The drug was given in divided doses by mouth. The initial dose was* 3 *grams daily, but this was increased to* 6 *grams daily for the last thirty-six hours (see text). Plasma corticosteroids level were measured at* 9.0. *a.m. The upper limit of the normal range at this time of day is shown by the horizontal dashed line. Urinary tetrahydro S excretion is shown by the shaded columns.*

*Post-mortem findings*

These confirm the diagnosis of an oat-cell carcinoma of the right upper lobe bronchus with widespread metastases in lymph nodes, liver, vertebral bodies and adrenal glands. The adrenal glands each weighed 18 grams, but some of this increase was due to extensive metastatic deposits. Marked hyperplasia of the zona fasciculata was seen on microscopy. The pituitary gland was normal in size and sections showed a normal distribution of cell types. The brain and meningse were normal but the femoral nerve showed some demyelinisation. The wasted voluntary muscle showed some patchy atrophy.

No corticotrophic activity was detected in extracts of the pituitary or primary tumour (Dr. Beryl M. A. Davies).

*Case 2.*

E. D., a man of 63, was admitted to hospital in April 1962. He complained of swelling of his face for three weeks which had been noticed both by himself and by his wife. A productive cough, wheezing and shortness of breath on exertion had been present for the same period. He had smoked 20 cigarettes daily for many years. Examination showed a healthy looking elderly man. His facial appearance was not suggestive of Cushing's syndrome but comparison with a photograph taken the previous year showed, however, that his face had indeed become fatter. There was no clubbing of the fingers. Inspiratory rales and a few rhonchi were present over the left upper chest. The liver edge was palpated 5 cm. below the costal margin and was hard and irregular. Multiple ectopic beats were present and his blood pressure was 210/110. The apex beat was displaced to the left and was left ventricular in character. A short apical systolic murmur was present. The jugular venous pressure was not increased and there was no oedema. The fundi were normal. There was no muscular weakness and all tendon reflexes were easily elicited. Mild glycosuria, without ketonuria, was present.

*Investigations*

Chest X-rays showed enlargement of the left hilar region suggestive of a bronchial carcinoma. X-rays of the pituitary fossa were normal. A liver biopsy showed infiltration by oat-cell carcinoma.

The fasting blood glucose level was 100 mg. per 100 ml. with a diabetic glucose tolerance curve. The blood urea was 36 mg. per 100 ml. and a hypokalaemic alkalosis was present (potassium 2·0, bicarbonate 35, sodium 142, and chlorides 80 mEq. per litre). The urinary potassium excretion on a normal ward diet was 25 mEq. per day. He was given an oral potassium supplement of 140 mEq. a day but the plasma potassium concentration failed to rise above 3·1 mEq. per litre.

*Adrenal function tests*

Free plasma corticosteroid levels were very high and ranged from 53 to 66 $\mu$g. per 100 ml. The normal diurnal rhythm was absent. Urinary 17-hydroxy-corticoids were also markedly elevated at 113 mg. per twenty-four hours. These results are recorded in Table I.

*Dexamethasone suppression test*

Dexamethasone was given by mouth in a dose of 2·0 mg. every six hours for forty-eight hours. This dosage did not produce any significant depression of adrenocortical function. Urinary 17-hydroxycorticoids were 109 mg. per twenty-four hours on the second day, and the plasma 11-O.H.C.S. level only fell from 65 to 57 $\mu$g. per 100 ml. (Fig. 2).

FIG. 2. *Response to dexamethasone (DX) and metapirone (MET) in Case 2. The drugs were given in divided doses by mouth. Dexamethasone was given in a dose of 8 mg. daily for forty-eight hours. Metapirone was given in a dose of 3 grams daily for a similar period (see text). Plasma corticosteroid levels were measured at 9.0 a.m. The upper limit of the normal range at this time of day is shown by the horizontal dashed line.*

*Response to Metapirone*

Metapirone was given by mouth in a dose of 500 mg. every four hours. After twenty-four hours on this drug the patient complained of general malaise and his blood pressure fell from 210/110 to 110/60. At this time his plasma 11-O.H.C.S. level had fallen from 65 to 15 μg. per 100 ml., and the fasting blood glucose level had fallen from 170 to 60 mg. per 100 ml. During the next twenty-four hours he became febrile (100°F) and extremely dyspnoeic. The fever and dyspnoea were thought to be due to left ventricular failure and broncho-pneumonia. The metapirone was discontinued and intravenous antibiotics, digoxin and prednisone were given. He improved over the next few days and the blood pressure and fasting blood glucose returned to their previous levels. There was no significant change in the plasma 17-O.H.C.S. level during metapirone administration but the urinary 17-hydroxycorticoids fell from 150 mg. to 63 mg. in twenty-four hours. They did not return to their original level when the drug was stopped. This discrepancy between plasma and urinary 17-hydroxycorticoid estimations may have been due to an alteration in the renal handling of conjugated steroids during this period, since the urine output dropped from 3·0 to 1·3 litres a day when metapirone was given. These changes are shown graphically in Figure 2.

*Response to hypophysectomy*

Hypophysectomy was carried out by Mr. J. Angell James through a Chiari (trans-sphenoidal) approach on 16th May 1962. The operation was covered with oral and intravenous prednisolone, and oral prednisolone was continued during the post-operative period. He appeared to be recovering fairly satisfactorily from this operation but his condition rapidly deteriorated on the sixth post-operative day and he died in left ventricular failure. Free plasma 11-O.H.C.S. levels during this time remained elevated and suggested that removal of his pituitary had had little effect on his increased adrenocortical activity (Table II).

TABLE II

Effect of Hypophysectomy on Morning Free Plasma 11-hydroxycorticoid Levels in Case 2 (μg. per 100 ml.)

| Pre-operative Levels | Post-Operative Day | | | |
|---|---|---|---|---|
| | 1 | 2 | 5 | 6 |
| 60·1–66·1 | 52·1±0·8 | 54·2±3·1 | 63·4±0·3 | 64·3±1·5 |

*Post-mortem findings*

These confirmed the diagnosis of an oat-cell carcinoma of the left lower lobe bronchus. A lung abscess was found distal to the tumour. Metastases were found in the mediastinal lymph nodes and liver. No metastases were found in the adrenal glands which were enlarged, the right weighing 19 and the left 16 grams respectively. Marked hyperplasia of the zona fasciculata was seen on microscopy. The pituitary fossa was empty except for a few fragments of anterior pituitary tissue in the floor of the fossa. It was estimated that they did not form more than 5 per cent of the whole pituitary gland.

*Plasma corticosterone estimations*

Plasma corticosterone levels were measured in both patients and are recorded in Table III. They are compared with similar estimations in three hospital patients without any endocrine abnormality and five patients with Cushing's syndrome and normal plasma electrolytes. Normal levels were found in all these patients.

TABLE III

Free Plasma Corticosterone Levels

| Patients | No. | Plasma 11-O.H.C.S. (µg. per 100 ml.) | |
|---|---|---|---|
| | | Total | Corticosterone |
| Normal hospital patients | 3 | 20·5–27·4 | 0·3–0·5 |
| Cushing's syndrome with normal electrolytes | 5 | 23·2–72·9 | 0–2·0 |
| Case 1 | | 74·4 | 1·4 |
| Case 2 | | 58·9 | 0·5 |

### DISCUSSION

Both patients had oat-cell carcinoma of the bronchus and an unexpected hypokalaemic alkalosis and glycosuria. Typical clinical features of Cushing's syndrome were absent, but further investigations demonstrated increased adrenocortical activity. Hyperplastic adrenals were found at post-mortem examination and there seems little doubt that these glands were secreting the excessive amounts of corticosteroids found in life. This bilateral adrenal hyperplasia has been found in most of the cases reported in the literature and appears to be independent of the presence of adrenal metastases. Our first patient had extensive adrenal metastases whilst none were found in the second patient.

### *Response to metapirone*

Metapirone inhibits 11 β-hydroxylation in the adrenal cortex, and 11-deoxycortisol (compound S) is secreted instead of cortisol (Liddle *et al.*, 1958; Jenkins *et al.*, 1958). The level of free plasma 11-hydroxycorticoids falls as a result of this metabolic block in the synthesis of cortisol. This fall stimulates the pituitary to secrete more corticotrophin unless the pituitary is already inhibited. A rise in the urinary excretion of 17-oxogenic and 17-hydroxycorticoids results. Much of this rise will be due to the excretion of tetrahydro-11-deoxycortisol (tetrahydro S), one of the main metabolites of 11-deoxycortisol. An excessive rise in these urinary metabolites should occur when metapirone is given to patients with uncomplicated Cushing's syndrome due to adrenal hyperplasia (Liddle *et al.*, 1959).

There was a marked fall in the free plasma 11-hydroxycorticoid levels in both our patients during metapirone administration. Their fasting blood glucose levels and blood pressure also fell, and the psychotic state of our first patient was temporarily relieved. There was therefore clear evidence that the secretion of adrenocortical hormones was reduced when metapirone was given. This suggests that it might be of value in reducing adrenocortical activity pre-operatively in patients with Cushing's syndrome caused by an autonomous adrenal tumour.

There was a fall in the urinary excretion of corticosteroids in both patients during metapirone administration instead of the anticipated rise. Urinary tetrahydro S was measured on alternate days in the first patient and accounted for 59 to 85 per cent of the total 17-oxogenic steroids excerted. The free plasma 17-hydroxycorticoid level fell in Case 1 and it is probable that the metapirone was blocking other enzymes besides 11 β-hydroxylase in this patient. This fall was not seen in Case 2.

Their responses to metapirone were unlike those seen in patients with uncomplicated Cushing's syndrome due to adrenal hyperplasia and can only be explained if their adrenals were functioning autonomously, if the source of corticotrophin was resistant to the usual stimulus of a falling plasma cortisol level, or if the secretion of corticotrophin or of adrenal steroids was already maximal. The latter explanation is the least likely since the free plasma 11-hydroxycorticoid level in our first patient rose to 115 $\mu$g. per 100 ml. a few hours before death. The results of the dexamethasone suppression test in the second patient provided further evidence in support of these conclusions. There was no significant depression of the urinary 17-hydroxycorticoids or free plasma 11-hydroxycorticoid level, when dexamethasone was given in a dose of 2 mg. six-hourly for forty-eight hours. Liddle (1960) has shown that this amount of dexamethasone will produce a significant decrease in the urinary 17-hydroxycorticoids in uncomplicated cases of Cushing's syndrome due to adrenal hyperplasia. No decrease is seen in patients with autonomous adrenal tumours.

*Results of hypophysectomy*

Hypophysectomy was carried out in the second patient in the hope that it might slow down the rate of growth of the tumour, already known to be inoperable, whilst correcting the endocrine disturbance. Prednisolone was used to cover and follow the operation since this steroid is not measured by the fluorimetric method of Mattingly. As a result endogenous plasma 11-hydroxycorticoid levels could be followed post-operatively.

There was no significant fall in these levels after hypophysectomy and it seems fairly certain that the excessive adrenocortical activity in this patient was independent of anterior pituitary function. The sella appeared to be empty at post-mortem examination. Histological examination did show a small amount of anterior pituitary tissue in the floor of the fossa but it is unlikely that this could have secreted enough corticotrophin to have maintained adrenocortical function at its previous high level.

Our findings in these two patients are compatible with the hypothesis that the excessive adrenocortical activity was independent of pituitary function and was due to the secretion of a corticotrophin-like substance by the tumour cells. The same conclusion was reached by Hudson and Evans (1962) from their studies on a man of 64 with an oat-cell bronchial carcinoma and excessive adrenocortical activity.

Dr. Beryl Davies did not find any corticotrophic activity in extracts of the primary tumour from our first patient, but this tissue was not obtained until some time after death. Christy (1961) however found corticotrophic activity in the plasma of two patients whose adrenocortical hyperplasia followed the appearance of a pulmonary neoplasm. Corticotrophic-like activity was found later in a metastasis from one of the patients (Holub and Katz, 1961). Bornstein *et al.* (1961), also found an elevated plasma cortico-trophin level in a patient with an oat-cell carcinoma of the trachea associated with adrenocortical hyperfunction. Recently, Meador *et al.* (1962), have reported the finding of a corticotrophin-like material in the plasma, primary tumour, and metastases of five patients with extra-adrenal carcinomas associated with excessive adrenocortical activity. The pituitary corticotrophin content, on the other hand, was abnormally low. All the signs of adrenocortical hyperfunction, including the hypokalaemia, were corrected by bilateral adrenalectomy in two of their patients.

*Potassium metabolism*

Both our patients had a hypokalaemic alkalosis with excessive renal loss of potassium. Although this appears to be a common finding in these patients with adrenocortical hyperfunction and extra-adrenal carcinomas it is not confined to them. Christy and

Laragh (1961) studied a group of forty patients with Cushing's syndrome and found a hypokalaemic alkalosis in twelve. Only three of these twelve patients had associated neoplasms outside the adrenals or pituitary. There was, however, a significantly higher mean plasma 17-hydroxycorticoid level of 55 $\mu$g. per 100 ml. in the hypokalaemic patients compared to the mean level of 30 $\mu$g. per 100 ml. in the patients with no electrolyte abnormality. In addition, the urinary steroid values tended to be higher in the hypokalaemic patients. They suggested that the cause of the potassium depletion and metabolic alkalosis in Cushing's syndrome, irrespective of aetiology, was the grossly exaggerated secretion of cortisol rather than the oversecretion of aldosterone.

Aldosterone secretion was not measured in either of our patients, but normal urinary levels and normal secretion rates have been found sufficiently often to exclude hyperaldosteronism as a common cause for this metabolic abnormality in Cushing's syndrome. On the other hand, the cortisol secretion rate was very high in our first patient and high plasma and urinary corticosteroid levels were found in both.

The possibility that the electrolyte abnormality is due to the secretion of some hormone other than cortisol is not excluded by our findings. For example, Mader and Iseri (1955) found an excessive amount of a corticosterone-like steroid in the urine of a patient with an adrenocortical tumour who present with hypertension and a hypokalaemic alkalosis. Excessive amounts of corticosterone were extracted from the tumour. There was no evidence of excessive cortisol secretion in their patient.

Normal free plasma corticosterone levels were found in our two patients. The method used can be criticised for its lack of specificity, but the levels found in the other patients who had normal plasma electrolytes are in good agreement with those found in normal subjects after chromatographic separation of the plasma corticosteroids. Brooks *et al.* (1960) found no corticosterone in the plasma of another patient with Cushing's syndrome and severe hypokalaemic alkalosis even after corticotrophin stimulation.

It is therefore unlikely that the excessive secretion of corticosterone was responsible for the hypokalaemic alkalosis in our patients, but it is conceivable that they were secreting some other hormone, at present unidentified, which has a profound effect on potassium metabolism. Ross (1959) has reported a syndrome, identical with that described by Conn, in a boy of 13 who had a negligible aldosterone secretion rate. An abnormal steroid pattern in the urine was found on paper chromatography.

### SUMMARY

Two further patients with oat-cell carcinoma of the bronchus and greatly increased adrenocortical activity have been described. The response to metapirone was abnormal in both patients and suggested that their adrenocortical hyperfunction was independent of the pituitary. This was confirmed by the effect of hypophysectomy in one patient. It is probable that their adrenals were being stimulated excessively by a corticotrophin-like substance secreted by the tumour cells.

A temporary remission of their endocrine disorder was produced by metapirone, and this was associated with a marked fall in their free plasma 11-hydroxycorticoid levels.

Hypokalaemic alkalosis and excessive renal loss of potassium were present in both patients. The cause of this electrolyte abnormality in Cushing's syndrome is discussed.

### *Acknowledgements*

We are grateful to Dr. C. L. Cope, Dr. G. K. McGowan, and Professor C. Bruce Perry for their help and advice in the preparation of this paper. We are indebted to Mr. J. Angell James and Dr. P. Hugh-Jones for permission to publish clinical details; to Dr. Beryl M. A. Davies, Dr. C. Pollock, Dr. A. L. Taylor, and the Department of Chemical Pathology, Hammersmith Hospital, for their help in studying these patients; and to Upjohn Ltd., for a generous supply of pure cortisol and coticosterone, and Ciba Laboratories, Ltd. for the metapirone.

### REFERENCES

Allott, E. M., and Skelton, M. O. (1960). *Lancet*, **2**, 278.
Bagshawe, K. D. (1960). *Lancet*, **2**, 284.
Bornstein, P., Nolan, J. P. and Bernanke, D. *New Engl. J. Med.*, 264, 363.
Brooks, R. V., McSwiney, R. R., Mattingly, D. and Prunty, F. T. G. (1960). *J. Endocrin.*, **19**, 366.
Bush, I. E. and Sandberg, A. A. (1953). *J. biol. Chem.*, 205, 783.
Christy, N. P. (1961). *Lancet*, **1**, 85.
Christy, N. P. and Laragh, J. H. (1961). *New Eng. J. Med.*, 265, 1083.
Holub, D. A. and Katz, F. H. (1961). *Clin. Res.*, **9**, 194.
Hudson, B. and Evans, J. (1962). *J. clin. Endocrin.*, **22**, 494.
Jenkins, J. S., Meakin, J. W., Nelson, D. H. and Thorn, G. W., *Science*, 128, 478.
Liddle, G. W. (1960). *J. clin. Endocrin.*, **20**, 1539.
Liddle, G. W., Island, D., Lance, E. M. and Harris, A. P. (1958). *J. clin. Endocrin.*, **18**, 906.
Liddle, G. W., Estep, H. L., Kendall, J. W., Williams, W. C. and Townes, A. S. (1959). *J. clin. Endocrin.*, **19**, 875.
Mader, I. J. and Iseri, L. T. (1955). *Amer. J. Med.*, **19**, 976.
Mattingly, D. (1962). *J. clin. Path.*, **15**, 374.
Meador, C. K., Liddle, G. W., Island, D., Nicholson, W. E., Lucas, C. P., Nuckton, J. G. and Luetscher, J. A. (1962). *J. clin. Endocrin.*, **22**, 693.
Ross, E. J. (1959). *Proc. R. Soc. Med.*, **52**, 1056.

## Figures and Tables

**Fig. 1. f1:**
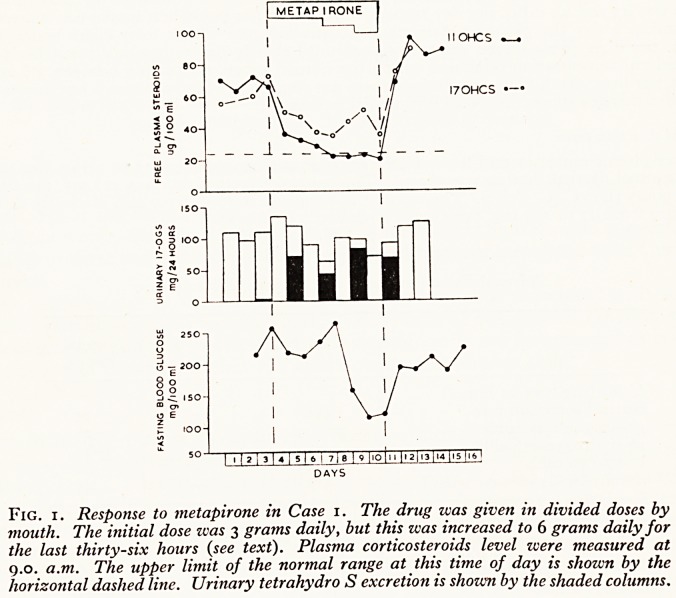


**Fig. 2. f2:**